# Screening, prevalence, treatment and control of kidney disease in patients with type 1 and type 2 diabetes in low-to-middle-income countries (2005–2017): the International Diabetes Management Practices Study (IDMPS)

**DOI:** 10.1007/s00125-021-05406-6

**Published:** 2021-02-16

**Authors:** Jean Claude Mbanya, Pablo Aschner, Juan J. Gagliardino, Hasan İlkova, Fernando Lavalle, Ambady Ramachandran, Jean-Marc Chantelot, Juliana C. N. Chan

**Affiliations:** 1 Biotechnology Center, Doctoral School of Life Sciences, Health and Environment, University of Yaoundé I, Yaoundé, Cameroon; 2 Department of Medicine and Specialties, Faculty of Medicine and Biomedical Sciences, University of Yaoundé I, Yaoundé, Cameroon; 3 Javeriana University School of Medicine, Bogotá, Colombia; 4 San Ignacio University Hospital, Bogotá, Colombia; 5 CENEXA (UNLP-CONICET-CEAS CICPBA), School of Medicine UNLP, La Plata, Argentina; 6 Istanbul University, Istanbul, Turkey; 7 Facultad de Medicina de la Universidad Autónoma de Nuevo León, Nuevo León, Mexico; 8 India Diabetes Research Foundation, Dr. A. Ramachandran’s Diabetes Hospitals, Chennai, India; 9 Sanofi, Paris, France; 10 Department of Medicine and Therapeutics, Faculty of Medicine, The Chinese University of Hong Kong, Prince of Wales Hospital, Hong Kong SAR, China; 11 Hong Kong Institute of Diabetes, The Chinese University of Hong Kong, Prince of Wales Hospital, Hong Kong SAR, China; 12 Obesity and Li Ka Shing Institute of Health Sciences, Faculty of Medicine, The Chinese University of Hong Kong, Prince of Wales Hospital, Hong Kong SAR, China

**Keywords:** Kidney disease, Low-and-middle-income countries, Microalbuminuria/proteinuria, Real-world study, Screening, Type 1 diabetes, Type 2 diabetes

## Abstract

**Aims/hypothesis:**

Diabetes is the leading cause of kidney disease worldwide. There is limited information on screening, treatment and control of kidney disease in patients with diabetes in low-to-middle-income countries (LMICs).

**Methods:**

The International Diabetes Management Practices Study is an ongoing, non-interventional study of clinical profiles and practices among patients receiving outpatient care mainly by internal medicine physicians and endocrinologists in LMICs. We examined screening, prevalence, treatment and control of kidney disease across seven waves (W) of data collection between 2005 and 2017.

**Results:**

Among 15,079 patients with type 1 and 66,088 patients with type 2 diabetes, screening for kidney disease increased between W2 and W3 followed by a plateau (type 1 diabetes: W2, 73.7%; W3, 84.1%; W7, 83.4%; type 2 diabetes: W2, 65.1%; W3, 82.6%; W7, 86.2%). There were also decreasing proportions of patients with microalbuminuria (type 1 diabetes: W1, 27.1%; W3, 14.7%; W7, 13.8%; type 2 diabetes: W1, 24.5%; W3, 12.6%; W7, 11.9%) and proteinuria (type 1 diabetes: W1, 14.2%; W3, 8.7%; W7, 8.2%; type 2 diabetes: W1, 15.6%; W3, 9.3%; W7, 7.6%). Fewer patients were reported as receiving dialysis for both type 1 diabetes (W2, 1.4%; W7, 0.3%) and type 2 diabetes (W2, 0.9%; W7, 0.2%) over time. While there was no change in mean HbA_1c_ or prevalence of diagnosed hypertension (type 1 diabetes: W1, 22.7%; W7, 19.9%; type 2 diabetes: W1, 60.9%; W7, 66.2%), the use of statins had increased among patients diagnosed with dyslipidaemia (type 1 diabetes: W1, 77.7%; W7, 90.7%; type 2 diabetes: W1, 78.6%; W7, 94.7%). Angiotensin II receptor blockers (type 1 diabetes: W1, 18.0%; W7, 30.6%; type 2 diabetes: W1, 24.2%; W7, 43.6%) were increasingly used over ACE inhibitors after W1 (type 1 diabetes: W1, 65.0%; W7, 55.9%; type 2 diabetes: W1, 55.7%, W7, 41.1%) among patients diagnosed with hypertension.

**Conclusions/interpretation:**

In LMICs, real-world data suggest improvement in screening and treatment for kidney disease in patients with type 1 and type 2 diabetes attending non-nephrology clinics. This was accompanied by decreasing proportions of patients with microalbuminuria and proteinuria, with fewer patients who reported receiving dialysis over a 12-year period.

**Graphical abstract:**

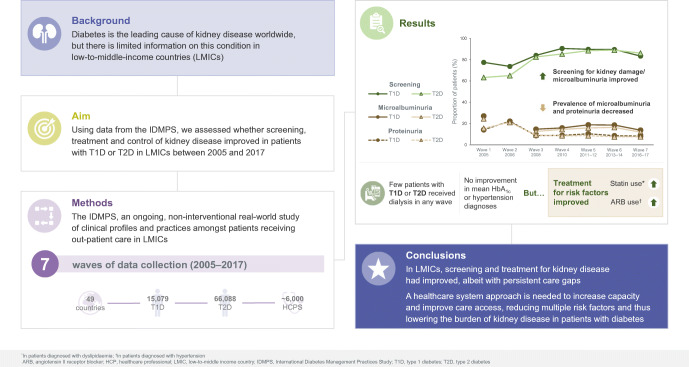

**Supplementary Information:**

The online version contains peer-reviewed but unedited supplementary material available at 10.1007/s00125-021-05406-6.

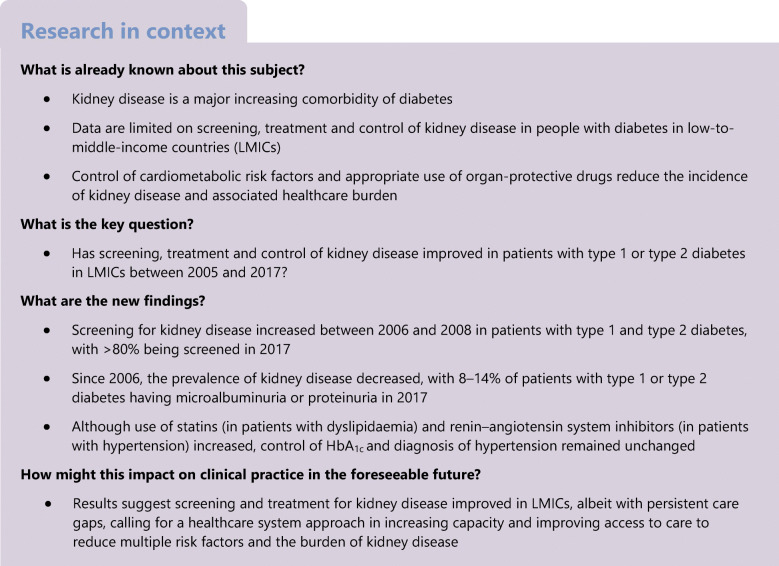



## Introduction

The global burden of kidney disease is rising along with an increasing prevalence of diabetes, the latter being a leading cause of kidney disease. Between 1990 and 2016, the global prevalence of chronic kidney disease (CKD) had increased by 87%, from 147.6 million to 275.9 million, while deaths due to CKD increased by 98%, from 0.6 million to 1.2 million [1]. In approximately 40% of patients with CKD, the CKD is directly attributed to diabetes, [1] which often coexists with other risk factors, such as hypertension [2]. In the USA National Health and Nutrition Examination Survey (NHANES) conducted between 2009 and 2014, approximately 25% of patients with diabetes had CKD [3]. Development of CKD increases the risk of hypoglycaemia and treatment complexity in patients with diabetes [4, 5]. Additionally, CKD and diabetes are independent risk factors for CVD [6, 7].

Regular screening to detect kidney disease can prompt early intervention which can reduce the incidence of end-stage kidney disease (ESKD) and healthcare costs, and increase ESKD-free survival [8, 9]. The US National Kidney Foundation recommend that patients with diabetes should undergo screening for kidney disease annually, beginning 5 years after diagnosis of type 1 diabetes and at diagnosis of type 2 diabetes [10]. Annual screening should include measurements of urinary albumin/creatinine ratio (ACR) to detect microalbuminuria and serum creatinine for calculation of eGFR [10]. The National Institute for Health and Care Excellence in the UK recommends increased frequency of screening in patients with or at risk of CKD, or with worsening kidney function (based on eGFR and/or ACR measurements) [11].

The prevalence and outcomes of kidney disease have strong socioeconomic determinants. The sociodemographic index of a country is based on average income per person, educational attainment and total fertility rate. In countries with a high sociodemographic index, the age-standardised prevalence of CKD has been shown to be lower than in countries with a low sociodemographic index [1]. In this global epidemic of diabetes where low-to-middle-income countries (LMICs) are hit hardest with the dual burden of diabetes and kidney disease [2], there are limited data on the pattern of screening, prevalence, treatment and control of kidney disease for both type 1 and type 2 diabetes to inform practice and policies.

The International Diabetes Management Practices Study (IDMPS) is an ongoing worldwide observational survey that describes the clinical profiles and management of patients receiving outpatient care mainly by internal medicine physicians and endocrinologists in LMICs [12]. Data were collected in seven successive waves between 2005 (Wave [W]1) and 2017 (W7). In this analysis, we examined screening, prevalence, treatment and control of kidney disease (microalbuminuria, proteinuria) in patients with type 1 or type 2 diabetes during a 12 year period.

## Methods

### Study design

The IDMPS is an ongoing, non-interventional international study documenting diabetes care practices involving more than 80,000 patients with diabetes managed by over 6000 physicians living across 49 LMICs. Between 2005 and 2017, real-world data were collected in seven individual waves conducted in different regions and countries (electronic supplementary material [ESM] Table 1). Each wave comprised a 2 week cross-sectional study when consecutive patients were recruited from participating clinics.

Primary care and specialist physicians (i.e. internal medicine specialists, diabetologists, endocrinologists) who were familiar with the use of insulin were invited to enrol their first five patients with type 1 diabetes and ten patients with type 2 diabetes (male or female) who attended their clinics during the 2 week recruitment period. Clinical data were collected and completed by the physicians using standardised case report forms (CRFs). Physicians recorded whether the patient had been screened for kidney disease within the 12 months prior to the study visit, and the frequency of screening. Details of setting/physicians who performed the screening were not recorded.

The first patient in W1 was enrolled on 31 January 2004 and the last patient completed W7 on 12 October 2016. The study design and reporting format were in accordance with STROBE (STrengthening of the Reporting of OBservational studies in Epidemiology) guidelines. Ethical approval was obtained from institutional review boards in each country and the study was conducted in accordance with the Declaration of Helsinki. All patients provided written informed consent.

### Participants

Patients with type 1 or type 2 diabetes over the country-specific adult legal age were eligible to participate. Exclusion criteria included concomitant participation in another clinical study, participation in a previous wave of IDMPS, or current receipt of temporary insulin therapy due to gestational diabetes, pancreatic cancer or surgery.

### Outcome measurements

The primary outcome of this analysis was the proportion of patients who underwent screening for kidney disease within the 12 months prior to each study visit, and the prevalence of kidney disease over a 12 year period. Participating physicians completed the CRFs using simple coding of Yes/No at their discretion. Outcomes included the presence of microalbuminuria (determined by laboratory measurement or desktop machines prior to this study) or proteinuria (determined by dipstick method prior to this study). Urinary ACR values were not recorded and data on eGFR were collected in W7 only with CKD (stage 3) defined as eGFR <60 ml min^−1^ [1.73 m]^−2^. Additionally, physicians were asked to document diagnosed hypertension and dyslipidaemia, reported dialysis treatment and presence of microvascular (e.g. retinopathy, visual impairment and sensory neuropathy) and macrovascular (e.g. stroke, ischaemic heart disease, myocardial infarction, acute coronary syndrome and revascularisation procedures) complications. The last HbA_1c_ value and BP in the last 12 months, body weight/height measured during the clinic visit, as well as current treatments, were recorded.

### Statistical analysis

The sample size of IDMPS was determined for each country based on the primary objective of IDMPS, which aimed to assess the therapeutic management of patients with type 2 diabetes and establish the proportion of those patients with insulin-treated type 2 diabetes, assuming that insulin was the least prescribed therapy for type 2 diabetes. For an estimated proportion of 20% with a precision of 4% and a 95% CI of 16–24%, a sample size of 384 patients would be required from each country. For a prevalence of 5% with a precision of 1% and a 95% CI of 4–6%, a sample size of 1825 patients would be required. The actual number of patients recruited from each country varied according to the estimated prevalence of patients with insulin-treated type 2 diabetes in that country (ESM Methods). Physicians experienced in prescribing insulin therapy were selected randomly after stratification based on specialty (primary care doctors or endocrinologists). The number of participating physicians recruited was based on the country-specific estimated patient sample size required.

The eligible population comprised all patients with no missing data concerning diabetes treatment. Missing data were not imputed, except for day or month of birth, which were set to ‘15’ or ‘June’, respectively. Categorical variables were presented using counts and percentages and continuous variables as means and SDs. Due to the descriptive nature of the study and the fact that no formal hypothesis was tested, descriptive analyses were performed with no comparative analyses or *p* values.

## Results

### Patient characteristics

A total of 15,079 patients with type 1 diabetes and 66,088 patients with type 2 diabetes were included in the seven waves of data collection. Patient demographics and clinical characteristics are described in Table 1. Among patients with type 2 diabetes, 42,171 (63.8%) were treated with oral glucose-lowering drugs (OGLDs) alone, 7566 with insulin alone (11.4%) and 14,529 (22.0%) with OGLDs plus insulin. The distribution of participation in healthy diet and exercise plans and use of OGLDs and insulin in each patient group is shown in ESM Table 2. Patients with type 1 diabetes were more likely to follow a healthy diet and exercise plan than patients with type 2 diabetes. Metformin and sulfonylureas were the most common OGLDs. The use of glucagon-like peptide 1 receptor agonists (GLP-1 RA) was recorded from W4 onwards and less than 3.0% of patients received this drug between W4 and W7. The use of sodium–glucose cotransporter-2 inhibitors (SGLT2i) was captured only in W7. A total of five patients (3.4%, *n* = 149) with type 1 diabetes and 21 patients (1.0%, *n* = 2125) with type 2 diabetes were treated with an SGLT2i. Insulin was administered to every patient with type 1 diabetes, with 52–68% treated with a basal–bolus regimen and 17–24% treated with a premix insulin regimen. In patients with type 2 diabetes, insulin use varied between 30% and 41% across W1 to W7. In patients with type 1 or type 2 diabetes, mean HbA_1c_ was similar across all waves, and glycaemic target achievement (<53 mmol/mol [<7%]) remained suboptimal (20.7–38.0%) over time (Table 1). Mean BMI was similar over time in type 1 diabetes (W1, 23.4 [SD 3.7] kg/m^2^; W7, 24.7 [4.6] kg/m^2^) and increased over time with type 2 diabetes (W1, 27.1 [4.8] kg/m^2^; W7, 29.8 [5.5] kg/m^2^) with a mean difference (95% CI) of 2.68 (2.52, 2.85) kg/m^2^ (Table 1).

**Table 1:** Demographic and clinical characteristics of patients with type 1 and type 2 diabetes

Characteristic	Wave 1 2005	Wave 2 2006	Wave 3 2008	Wave 4 2010	Wave 5 2011–12	Wave 6 2013–14	Wave 7 2016–17
Type 1 diabetes (*N* = 15,079)	(*n* = 1845)	(*n* = 3507)	(*n* = 2337)	(*n* = 958)	(*n* = 2789)	(*n* = 1643)	(*n* = 2000)
Age, years	36.42 ± 13.98	35.24 ± 13.75	35.44 ± 14.72	34.89 ± 14.78	33.73 ± 13.02	33.88 ± 12.54	33.97 ± 12.32
Male	923 (50.4)	1642 (48.3)	1130 (49.2)	444 (46.7)	1366 (49.0)	805 (49.0)	976 (48.8)
BMI, kg/m^2^	23.41 ± 3.72	24.20 ± 4.48	24.36 ± 4.56	25.33 ± 4.82	24.18 ± 4.66	24.69 ± 4.58	24.73 ± 4.61
Systolic BP, mmHg	121.99 ± 17.07	121.26 ± 16.46	119.42 ± 16.04	118.84 ± 13.92	120.36 ± 14.90	121.55 ± 15.81	121.07 ± 14.56
Diastolic BP, mmHg	75.48 ± 9.55	74.93 ± 9.85	73.89 ± 9.56	74.84 ± 9.35	75.00 ± 9.44	75.67 ± 9.46	75.31 ± 9.42
Duration of diabetes, years	11.44 ± 9.16	11.62 ± 9.37	12.31 ± 9.68	13.38 ± 10.42	11.79 ± 9.23	12.00 ± 9.18	13.05 ± 9.88
Last known HbA_1c_,							
mmol/mol	67.20 ± 21.64	69.49 ± 23.94	67.31 ± 22.63	68.73 ± 22.63	68.84 ± 22.63	68.51 ± 20.44	68.18 ± 20.55
%	8.30 ± 1.98	8.51 ± 2.19	8.31 ± 2.07	8.44 ± 2.07	8.45 ± 2.07	8.42 ± 1.87	8.39 ± 1.88
HbA_1c_ <53 mmol/mol (<7%)	349 (25.5)	637 (23.1)	487 (25.7)	193 (22.9)	565 (22.7)	312 (20.7)	403 (21.8)
Microalbuminuria	400 (27.1)	NA	344 (14.7)	152 (15.9)	524 (18.8)	296 (18.6)	267 (13.8)
Proteinuria	239 (14.2)	704 (22.2)	204 (8.7)	85 (8.9)	290 (10.4)	137 (8.6)	159 (8.2)
CKD	NA	NA	NA	NA	NA	NA	159 (10.5)
Dialysis	NA	49 (1.4)	34 (1.5)	14 (1.5)	24 (0.9)	8 (0.5)	6 (0.3)
Microvascular complications^a^	958 (60.1)	1505 (47.0)	829 (35.5)	370 (38.6)	1336 (49.4)	903 (56.8)	924 (47.7)
Macrovascular complications^b^	220 (12.4)	307 (9.2)	184 (7.9)	90 (9.4)	275 (10.2)	172 (10.8)	115 (5.9)
Type 2 diabetes (*N* = 66,088)	(*n* = 9918)	(*n* = 17,232)	(*n* = 12,210)	(*n* = 5343)	(*n* = 9603)	(*n* = 5479)	(*n* = 6303)
Age, years	58.05 ± 11.51	58.18 ± 11.79	57.70 ± 11.84	58.42 ± 11.94	57.58 ± 11.20	57.33 ± 10.68	57.19 ± 11.09
Male	4749 (48.1)	7993 (47.8)	5490 (45.9)	2421 (45.7)	4486 (46.7)	2431 (44.4)	3012 (47.8)
BMI, kg/m^2^	27.12 ± 4.80	28.45 ± 5.26	28.97 ± 5.53	29.75 ± 5.49	29.60 ± 5.50	30.20 ± 5.67	29.80 ± 5.53
Systolic BP, mmHg	134.47 ± 19.26	133.32 ± 18.57	129.87 ± 17.19	129.66 ± 17.48	133.21 ± 17.20	133.75 ± 17.02	134.07 ± 16.74
Diastolic BP, mmHg	80.81 ± 10.37	79.75 ± 10.52	78.45 ± 9.79	78.52 ± 10.05	80.45 ± 10.03	80.67 ± 10.32	80.79 ± 9.86
Duration of diabetes, years	8.25 ± 7.08	8.55 ± 7.83	8.83 ± 7.74	9.09 ± 8.10	8.71 ± 7.32	9.26 ± 7.13	9.76 ± 7.38
Last known HbA_1c_,							
mmol/mol	61.51 ± 19.46	63.15 ± 21.10	62.28 ± 21.31	62.61 ± 20.22	64.36 ± 20.99	64.14 ± 19.46	64.57 ± 20.66
%	7.78 ± 1.78	7.93 ± 1.93	7.85 ± 1.95	7.88 ± 1.85	8.04 ± 1.92	8.02 ± 1.78	8.06 ± 1.89
HbA_1c_ <53 mmol/mol (<7%)	2168 (36.0)	4275 (35.2)	3446 (38.0)	1625 (37.1)	2658 (31.8)	1449 (29.4)	1716 (30.1)
Microalbuminuria	1713 (24.5)	NA	1540 (12.6)	773 (14.5)	1530 (15.9)	870 (16.4)	735 (11.9)
Proteinuria	1331 (15.6)	3128 (21.1)	1130 (9.3)	453 (8.5)	829 (8.6)	400 (7.5)	471 (7.6)
CKD	NA	NA	NA	NA	NA	NA	1058 (21.6)
Dialysis	NA	146 (0.9)	128 (1.0)	51 (1.0)	35 (0.4)	14 (0.3)	15 (0.2)
Microvascular complications^a^	5199 (65.1)	7380 (49.2)	4732 (38.8)	2271 (42.5)	4583 (49.6)	2895 (54.5)	2988 (48.4)
Macrovascular complications^b^	2560 (28.1)	3929 (24.4)	2345 (19.2)	1088 (20.4)	2103 (22.8)	1311 (24.7)	990 (16.0)

Values are mean ± SD or *n* (%). All complications were reported by physicians (Y/N)

Percentages were calculated for patients with available data; these varied by each category/wave

CKD defined as eGFR <60 ml min^-1^ [1.73 m]^-2^

^a^Retinopathy, visual impairment, sensory neuropathy, microalbuminuria, proteinuria and dialysis

^b^Stroke and ischaemic heart disease, myocardial infarction or acute coronary syndrome or history of revascularisation

NA, not available

### Screening for kidney disease

**Fig. 1:**
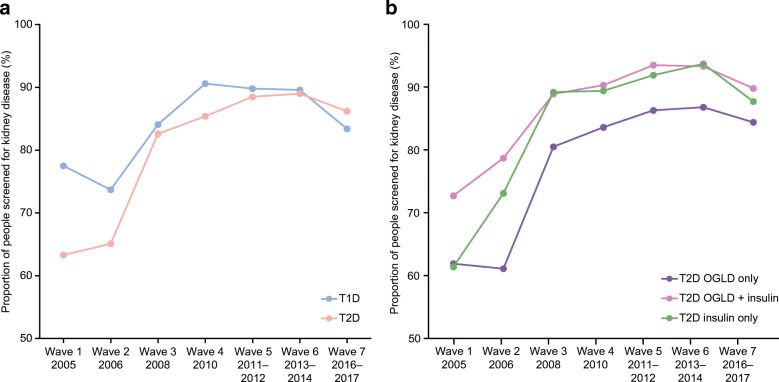
Screening for kidney disease in (**a**) patients with type 1 and type 2 diabetes over time and (**b**) patients with type 2 diabetes divided by therapy type. Percentages were calculated for patients with available data; these varied by each category/wave. T1D, type 1 diabetes; T2D, type 2 diabetes

Between W2 and W3 (2006 and 2008), screening for kidney disease markedly increased followed by a plateau thereafter in both patients with type 1 and type 2 diabetes (Fig. 1a). A similar increase was found in patients with type 2 diabetes when divided by therapy type (Fig. 1b). A greater proportion of patients with type 1 diabetes were screened vs those with type 2 diabetes. Among patients with type 2 diabetes, those treated with insulin were more likely to be screened. By W7, over 80% of patients in both groups had undergone screening in the previous 12 months.

**Fig. 2:**
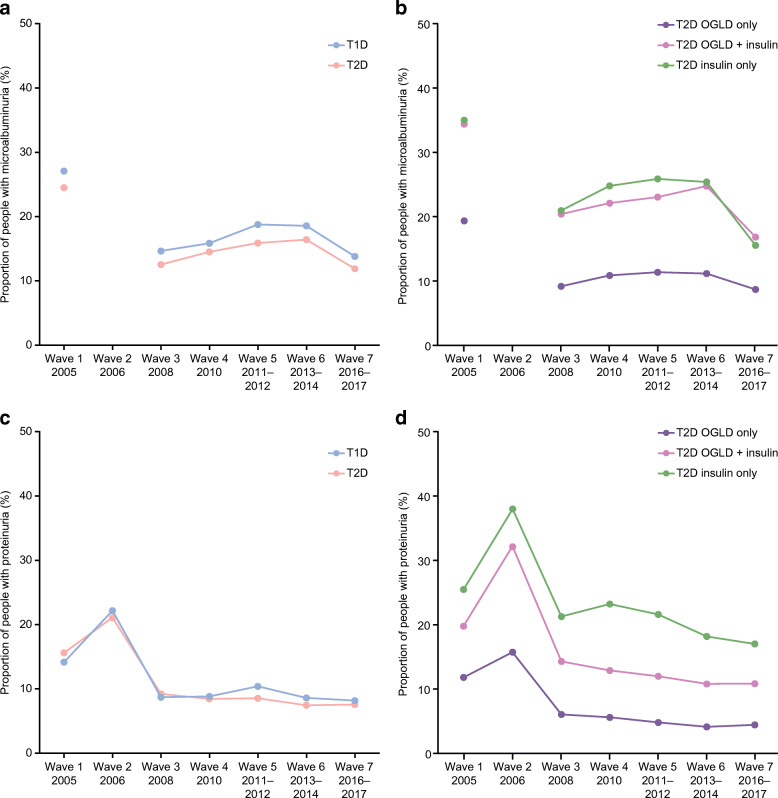
Microalbuminuria in patients with type 1 and type 2 diabetes (**a**) and in patients with type 2 diabetes divided by therapy type (**b**); proteinuria in patients with type 1 and type 2 diabetes (**c**) and in patients with type 2 diabetes divided by therapy type (**d**). Percentages were calculated for patients with available data; these varied by each category/wave. Microalbuminuria data were not available in W2. T1D, type 1 diabetes; T2D, type 2 diabetes

In both patients with type 1 and type 2 diabetes, the proportion with microalbuminuria/proteinuria decreased between W1 and W7 (2005–2017) with the greatest decrease observed between W1 and W3 (Fig. 2a, c). A similar pattern was seen in patients with type 2 diabetes irrespective of therapy type. Throughout the seven waves, patients with type 2 diabetes treated solely with OGLDs had the lowest proportion of microalbuminuria/proteinuria and those treated with insulin alone had the highest (Fig. 2b, d).

Overall, 0.2–1.5% of patients with type 1 or type 2 diabetes were recorded by the participating physicians (general practitioners, internal medicine specialists, endocrinologists or diabetologists) as requiring dialysis during all waves (Table 1). Based on eGFR data collected in W7, a greater proportion of patients with type 2 diabetes had CKD stage 3 (eGFR <60 ml min^−1^ [1.73 m]^−2^) than patients with type 1 diabetes (21.6% vs 10.5%, respectively) (Table 1 and ESM Table 3). An eGFR of <15 ml min^−1^ [1.73 m]^−2^, indicative of ESKD (CKD stage 5), was reported in 2.6% of patients with type 1 diabetes and 2.3% of patients with type 2 diabetes (ESM Table 3).

### Hypertension and associated treatments

The proportion of patients with diagnosed hypertension was similar over time in patients with type 1 diabetes (22.7% in W1; 19.9% in W7). The proportion of patients with type 2 diabetes diagnosed with hypertension increased over time (60.9% in W1; 66.2% in W7) (ESM Table 4). Among patients diagnosed with hypertension, the use of ACE inhibitors (ACEi) was highest in W1 (type 1 diabetes, 65.0%; type 2 diabetes, 55.7%) but had fallen by W7 (type 1 diabetes, 55.9%; type 2 diabetes, 41.1%). Increasing use of angiotensin II receptor blockers (ARBs) was evident in subsequent waves (type 1 diabetes, 18.0% in W1; 30.6% in W7; type 2 diabetes, 24.2% in W1; 43.6% in W7). During this period, statin use among patients diagnosed with dyslipidaemia also increased for patients with type 1 diabetes (W1, 77.7%; W7, 90.7%) and type 2 diabetes (W1, 78.6%; W7, 94.7%; ESM Table 4).

## Discussion

In this survey with real-world data from 81,167 patients with type 1 and type 2 diabetes recruited from 49 LMICs between 2005 and 2017, we have observed increased screening for kidney disease, with the greatest improvement occurring between 2005 and 2008. This was accompanied by declining proportions of patients with kidney disease (microalbuminuria, proteinuria or reported dialysis treatment). Of note, the prevalence of microalbuminuria, proteinuria, and microvascular and macrovascular complications decreased sharply between W1 (2005) and W3 (2008) and then plateaued; minor fluctuations were subsequently seen in the prevalence of microalbuminuria, although the prevalence of proteinuria remained low. While glycaemic control and diagnosis of hypertension did not improve over time, the use of statins among patients diagnosed with dyslipidaemia and renin–angiotensin system inhibitors (RASis) among patients diagnosed with hypertension increased. The renoprotective effects of RASis in type 1 and type 2 diabetes have been confirmed in large scale randomised clinical trials [13–15]. Despite these improvements, 12–14% of patients had microalbuminuria and approximately 8% had proteinuria in 2017. These care gaps call for further treatment intensification and improvement in self-management.

These results align with those from the 2019 International Society of Nephrology (ISN) Global Kidney Health Atlas which reported a global screening rate for kidney disease of 90–100% [16]. Among patients with diabetes, the estimated proportion of patients screened for kidney disease was 86% in LMICs, 95% in upper-middle-income countries and 98% in high-income countries [16].

In our analysis, patients with type 1 diabetes were more likely to be screened (74–91%) than those with type 2 diabetes (63–89%). Interestingly, patients with type 1 diabetes were more likely to follow a healthy diet than those with type 2 diabetes, suggesting possible care disparity between type 1 and type 2 diabetes. However, patients with type 2 diabetes treated with OGLDs plus insulin or insulin alone were more likely to have microalbuminuria than those with type 1 diabetes (Fig. 2a). This was possibly due to the older age and higher prevalence of diagnosed hypertension in patients with type 2 diabetes compared with those with type 1 diabetes. Among patients with type 2 diabetes, those treated with insulin were more likely to be screened than those who were not. This suggests that physicians recognise the high risk profile for patients treated with insulin, which is characterised by worse glycaemic control, more complications and longer disease duration vs those treated with OGLDs [17].

In 2017 (W7), when eGFR results were captured, approximately one in five patients with type 2 diabetes treated with OGLDs alone, one in three patients with insulin-treated type 2 diabetes and one in ten patients with type 1 diabetes had CKD stage 3 or above. In W7, 2–5% of patients had ESKD based on eGFR data. In comparison, 0.2–1.5% reported dialysis in all waves, suggesting possible under-reporting or non-commencement of dialysis despite the presence of ESKD.

In our results, the prevalence of microalbuminuria (type 1 diabetes: 13.8–27.1%; type 2 diabetes: 11.9–24.5%) was higher than that reported in a meta-analysis of 71 observational studies from 30 countries (predominantly high-income countries, with more than a third from the USA) [18]. In this meta-analysis, the prevalence of microalbuminuria was 1.3–3.8% in patients with type 1 diabetes and 3.8–12.7% in patients with type 2 diabetes [18]. However, differences in study design, populations and settings could lead to marked variations in these results. To this end, the proportions of patients with microalbuminuria/proteinuria in our study was similar to another cross-sectional study conducted in the USA between 2009 and 2014 with documented urinary ACR measurements [3].

Due to the silent nature of kidney disease, screening by blood and urine tests is necessary to inform medication use. Screening for CKD has been shown to reduce the incidence of ESKD and healthcare costs [8, 9]. By reducing multiple risk factors including high BP, lipids, HbA_1c_ and use of organ-protective drugs such as statins and RASis, the rate of decline of eGFR can be attenuated [8, 9]. In this light, the US National Kidney Foundation recommends the implementation of a country-wide screening programme of patients with diabetes to detect kidney disease at least once yearly using several diagnostic methods [10]. These screening programmes are low-cost strategies, compared with the high cost of dialysis, and have huge public health benefits [19, 20]. Given the high prevalence of kidney disease in LMICs, which have the least capacity to provide expensive treatment for ESKD including dialysis, increased investments to detect and treat kidney disease early should be a policy priority in LMICs [9].

Many patients in LMICs first present to clinical practice with advanced stages of kidney disease, sometimes requiring dialysis at first presentation [21]. In LMICs, governments often provide essential medical coverage mainly for acute illnesses or hospitalisation. In a survey of 130 countries affiliated with the ISN, none of the low-income and lower-middle-income countries reported implementation of a fully subsidised screening and care programme for patients with non-dialysis CKD [19]. Hence, despite the potential cardiovascular–renal protective effects of statins [22] and RASis [13, 14, 23], many patients had to pay out of pocket for these screening tests and preventive medications. In a cross-sectional survey from Asia conducted between 2007 and 2012 including LMICs (China, India, The Philippines and Vietnam) and high-income countries/areas (Hong Kong, South Korea and Taiwan), suboptimal control of cardiometabolic risk factors and low usage of statins and RASis were major risk factors for CKD [24].

In this analysis of real-world data, approximately 20% of patients with type 1 diabetes and approximately 60% of patients with type 2 diabetes were diagnosed with hypertension. Among these diagnosed patients, over 90% received antihypertensive therapies, including RASis. Over 77% of patients diagnosed with dyslipidaemia received statins across all waves. Both of these treatments have been shown to prevent or delay onset of CKD including ESKD and death in patients with type 2 diabetes [13, 14, 22, 23]. In a separate analysis of the IDMPS dataset, we reported declining proportions of patients with type 2 diabetes with microvascular and macrovascular complications accompanied by increasing use of statins and RASis (predominantly due to an increase in ARBs) (Ramachandran A, Lavalle F, Aschner P, et al, unpublished results).

Better control of BP and lipids is expected to reduce the incidence of CVD. However, ageing and increasing survival along with suboptimal glycaemic control and earlier onset of diabetes will continue to escalate the burden of CKD and ESKD, as reported in the USA [20]. Control of multiple risk factors, including optimising glycaemic and BP control, has been shown to reduce the risk of microvascular and macrovascular morbidity, ESKD and premature death [25, 26]. In our previous IDMPS reports, we reported suboptimal and worsening glycaemic control in patients with type 2 diabetes over a 12-year period, despite increasing prescriptions of insulin [17]. The use of GLP-1 RA and SGLT2i has been shown to confer cardiovascular–renal protection [27], although the use of these new medications was low in these LMICs. However, our previous IDMPS data indicated associations between good glycaemic control and self-monitoring of blood glucose and patient education, as well as reduced odds of complications and utilisation of healthcare resources [28–30]. Given the size of the problems of diabetes and kidney disease, policy and system changes are needed to raise physician awareness and maximise their efforts to reduce multiple risk factors while empowering patients to self-manage their diabetes and thus reduce the burden of kidney disease.

The strength of this study is the large sample size with global representation outside Western Europe and North America where data are needed to inform practice and policies. The main limitations include its observational nature with physician-reported performance of screening procedure without record of laboratory results. We did not record the setting and physicians who performed screening for kidney disease (e.g. general practitioners or specialists including endocrinologists or nephrologists) in the previous 12 months. The distribution of participating countries varied between waves, which might influence the trends over time, with fewer LMIC countries from Africa, Eurasia and the Middle East represented in the earlier waves. In many LMICs, patients may need to pay out of pocket for these screening tests which might be performed in other clinics or laboratories, making it logistically challenging to verify the results. We relied on physicians to report the presence of microvascular and macrovascular complications which might be influenced by recall bias, subjective interpretation or incomplete reporting. In 2015, the National Kidney Foundation guidelines recommended annual measurement of eGFR [10] which was included as a variable in W7. Prior to that, we used microalbuminuria and proteinuria as surrogates for kidney disease. We also do not have data regarding the use of ACEi and ARBs in patients without a diagnosis of hypertension. Due to the cross-sectional nature of each survey, we could not infer any causal relationship between the increasing prevalence of screening procedures and declining prevalence of kidney disease, and because of differences in healthcare systems and practices, these changes only reflected an overall pattern of variation between sites and waves. Self-selection also limited generalisability of these results since patients with advanced or suboptimally controlled diabetes may be more likely to seek medical advice, which might lead to overestimation of the prevalence of complications. On the other hand, since the majority of participating physicians were internal medicine physicians, family doctors and endocrinologists, this might explain the low prevalence of patients receiving dialysis reported in the present analysis, as these patients are more likely to be seen by nephrologists.

Briefly, in LMICs, between 2002 and 2017, increasing proportions of patients with type 1 and type 2 diabetes attending outpatient clinics in LMICs were screened for kidney disease. This was accompanied by increasing use of statins and RASis and decreasing prevalence of albuminuria/proteinuria over time. Despite these encouraging observations, in the most recent wave (2017), approximately 15% of patients had not been screened while 12–14% had microalbuminuria and approximately 8% had proteinuria. Given the persistently poor glycaemic control and lack of improvement in diagnosed hypertension, system-wide changes are needed to improve capacity and access for screening and control of multiple risk factors in order to reduce the burden of kidney disease in LMICs.

## Supplementary information

ESM (PDF 299 kb)

## Data Availability

Qualified researchers may request access to related study documents, which will be redacted to protect the privacy of study participants. Further details on Sanofi’s data sharing criteria, eligible studies and process for requesting access can be found at: https://www.clinicalstudydatarequest.com.

## Abbreviations

ACEi: ACE inhibitor
ACR: Albumin/creatinine ratio
ARB: Angiotensin II receptor blocker
CKD: Chronic kidney disease
CRF: Case report form
ESKD: End-stage kidney disease
GLP-1 RA: Glucagon-like peptide 1 receptor agonist
IDMPS: International Diabetes Management Practices Study
ISN: International Society of Nephrology
LMIC: Low-to-middle-income country
OGLD: Oral glucose-lowering drug
RASi: Renin–angiotensin system inhibitor
SGLT2i: Sodium–glucose cotransporter-2 inhibitor
W: Wave
