# Supplementary material for: Screening, prevalence, treatment and control of kidney disease in patients with type 1 and type 2 diabetes in low-to-middle-income countries (2005–2017): the International Diabetes Management Practices Study (IDMPS)
Source: Diabetologia. 2021 Feb 16;64(6):1246–55. doi: 10.1007/s00125-021-05406-6 (PMC8099838; doi:10.1007/s00125-021-05406-6)
Supplement: Supplementary file 1 — (PDF 299 kb) [file 125_2021_5406_MOESM1_ESM.pdf]

ESM methods

The sample size for IDMPS was determined on a country-basis, based on the primary objective of IDMPS, which was to assess the therapeutic management of patients with type 2 diabetes, and on the precision that was expected.

Based on the assumption that insulin was the least prescribed therapy for patients with type 2 diabetes, the sample size was determined to establish the prevalence of insulin treated patients with type 2 diabetes. The sample size was calculated to give an absolute precision of 20% of the estimated proportion of patients with type 2 diabetes receiving insulin and a confidence interval of 95%, using the following formula:

$n = p\ (1\text{-}p) \times (\varepsilon_\alpha / e)^2$

where:

n= the per country sample size

p = the estimated proportion of patients with T2D treated with insulin

ε_α = 1.96 for α = 5%

e = the absolute precision (20%) x p = the relative precision

Given this information, a computation table was determined that took into account the proportion of insulin treatments among all the prescriptions for patients with diabetes:

Proportion	Sample size
5%	1825
10%	864
15%	544
20%	384

For example, if in a given country, 10% of the patients receive insulin (p) with an absolute precision of 20%, the sample size (number of patients with type 2 diabetes to be recruited) would be 864 patients in this country for each wave.

ESM Table 1. Number of patients in participating countries and regions by wave

	Wave 1 / 2005	Wave 1 / 2005	Wave 2 / 2006	Wave 2 / 2006	Wave 3 / 2008	Wave 3 / 2008	Wave 4 / 2010	Wave 4 / 2010	Wave 5 / 2011–12	Wave 5 / 2011–12	Wave 6 / 2013–14	Wave 6 / 2013–14	Wave 7 / 2016–17	Wave 7 / 2016–17
	**T1D**	**T2D**	**T1D**	**T2D**	**T1D**	**T2D**	**T1D**	**T2D**	**T1D**	**T2D**	**T1D**	**T2D**	**T1D**	**T2D**
**Region/Country[a], n (%)**	**N=1845**	**N=9918**	**N=3507**	**N=17,232**	**N=2337**	**N=12,210**	**N=958**	**N=5343**	**N=2789**	**N=9603**	**N=1643**	**N=5479**	**N=2000**	**N=6303**
**Africa**	68[b] (3.7)	208 [b] (2.1)	824 (23.5)	2342 (13.6)	662 (28.3)	1721 (14.1)	116 (12.1)	314 (5.9)	741 (26.6)	2270 (23.6)	652 (39.7)	1761 (32.1)	788 (39.4)	2403 (38.1)
Algeria	0	0	217 (6.2)	456 (2.6)	194 (8.3)	469 (3.8)	0	0	222 (8.0)	514 (5.4)	79 (4.8)	196 (3.6)	82 (4.1)	198 (3.1)
Cameroon	0	0	0	0	0	0	0	0	38 (1.4)	524 (5.5)	92 (5.6)	199 (3.6)	77 (3.9)	211 (3.3)
Democratic Republic of Congo	0	0	0	0	0	0	0	0	0	0	0	0	25 (1.3)	50 (0.8)
Egypt	0	0	120 (3.4)	289 (1.7)	103 (4.4)	325 (2.7)	116 (12.1)	314 (5.9)	80 (2.9)	371 (3.9)	132 (8.0)	290 (5.3)	149 (7.5)	300 (4.8)
Ivory Coast	0	0	0	0	0	0	0	0	0	0	36 (2.2)	101 (1.8)	36 (1.8)	99 (1.6)
Kenya	0	0	0	0	0	0	0	0	0	0	0	0	7 (0.4)	187 (3.0)
Madagascar	0	0	0	0	0	0	0	0	0	0	0	0	25 (1.3)	50 (0.8)
Morocco	0	0	215 (6.1)	509 (3.0)	244 (10.4)	537 (4.4)	0	0	240 (8.6)	498 (5.2)	109 (6.6)	239 (4.4)	91 (4.6)	211 (3.3)
Nigeria	0	0	0	0	0	0	0	0	0	0	20 (1.2)	150 (2.7)	77 (3.9)	304 (4.8)
Senegal	0	0	0	0	0	0	0	0	25 (0.9)	50 (0.5)	30 (1.8)	96 (1.8)	43 (2.2)	101 (1.6)
South Africa	0	0	170 (4.8)	688 (4.0)	0	0	0	0	0	0	31 (1.9)	66 (1.2)	49 (2.5)	396 (6.3)
Tunisia	68 (3.7)	208 (2.1)	102 (2.9)	400 (2.3)	121 (5.2)	390 (3.2)	0	0	136 (4.9)	313 (3.3)	97 (5.9)	354 (6.5)	127 (6.4)	296 (4.7)
Zimbabwe	0	0	0	0	0	0	0	0	0	0	26 (1.6)	70 (1.3)	0	0
**Asia**	459 (24.9)	5392 (54.4)	618 (17.6)	4678 (27.1)	212 (9.1)	1604 (13.1)	0	0	428 (15.3)	1195 (12.4)	56 (3.4)	301 (5.5)	45 (2.3)	694 (11.0)
Bangladesh	5 (0.3)	520 (5.2)	0	0	0	0	0	0	0	0	0	80 (1.5)	1 (0.1)	199 (3.2)
China	63 (3.4)	548 (5.5)	102 (2.9)	540 (3.1)	140 (6.0)	521 (4.3)	0	0	0	0	0	0	0	0
Hong Kong	46 (2.5)	373 (3.8)	35 (1.0)	511 (3.0)	0	0	0	0	0	0	0	0	0	0
India	116 (6.3)	1639 (16.5)	181 (5.2)	854 (5.0)	0	0	0	0	368 (13.2)	994 (10.4)	0	0	44 (2.2)	495 (7.9)
Indonesia	15 (0.8)	708 (7.1)	12 (0.3)	674 (3.9)	0	0	0	0	0	0	0	0	0	0

Korea	151 (8.2)	845 (8.5)	0	0	0	0	0	0	0	0	0	0	0	0
Malaysia	0	0	64 (1.8)	472 (2.7)	39 (1.7)	561 (4.6)	0	0	0	0	0	0	0	0
Pakistan[b]	0	0	0	0	0	0	0	0	60 (2.2)	201 (2.1)	56 (3.4)	221 (4.0)	NA	NA
South Korea	0	0	142 (4.0)	807 (4.7)	0	0	0	0	0	0	0	0	0	0
Taiwan	42 (2.3)	382 (3.9)	39 (1.1)	398 (2.3)	0	0	0	0	0	0	0	0	0	0
Thailand	21 (1.1)	377 (3.8)	43 (1.2)	422 (2.4)	33 (1.4)	522 (4.3)	0	0	0	0	0	0	0	0
**Eurasia**	0	0	0	0	0	0	0	0	791 (28.4)	1834 (19.1)	713 (43.4)	1461 (26.7)	657 (32.9)	1330 (21.1)
Azerbaijan	0	0	0	0	0	0	0	0	0	0	28 (1.7)	122 (2.2)	0	0
Georgia	0	0	0	0	0	0	0	0	48 (1.7)	152 (1.6)	0	0	0	0
Kazakhstan	0	0	0	0	0	0	0	0	185 (6.6)	413 (4.3)	0	0	0	0
Russia	0	0	0	0	0	0	0	0	270 (9.7)	540 (5.6)	394 (24.0)	790 (14.4)	395 (19.8)	800 (12.7)
Ukraine	0	0	0	0	0	0	0	0	199 (7.1)	353 (3.7)	211 (12.8)	389 (7.1)	262 (13.1)	530 (8.4)
Uzbekistan	0	0	0	0	0	0	0	0	89 (3.2)	376 (3.9)	80 (4.9)	160 (2.9)	0	0
**Europe**	914 (49.5)	2605 (26.3)	807 (23.0)	2216 (12.9)	247 (10.6)	1158 (9.5)	50 (5.2)	603 (11.3)	115 (4.1)	842 (8.8)	0	0	0	0
Bosnia and Herzegovina	124 (6.7)	383 (3.9)	0	0	0	0	0	0	0	0	0	0	0	0
Bulgaria	233 (12.6)	539 (5.4)	244 (7.0)	532 (3.1)	0	0	0	0	0	0	0	0	0	0
Romania	371 (20.1)	747 (7.5)	356 (10.2)	742 (4.3)	0	0	0	0	0	0	0	0	0	0
Turkey	186 (10.1)	936 (9.4)	207 (5.9)	942 (5.5)	247 (10.6)	1158 (9.5)	50 (5.2)	603 (11.3)	115 (4.1)	842 (8.8)	0	0	0	0
**Latin America**	404 (21.9)	1713 (17.3)	848 (24.2)	6090 (35.3)	854 (36.5)	5704 (46.7)	587 (61.3)	3265 (61.1)	429 (15.4)	1397 (14.5)	0	0	0	0
Argentina	199 (10.8)	438 (4.4)	235 (6.7)	658 (3.8)	206 (8.8)	646 (5.3)	190 (19.8)	492 (9.2)	168 (6.0)	448 (4.7)	0	0	0	0
Chile	0	0	2 (0.1)	771 (4.5)	117 (5.0)	732 (6.0)	0	0	0	0	0	0	0	0
Columbia	103 (5.6)	577 (5.8)	64 (1.8)	1094 (6.3)	140 (6.0)	755 (6.2)	143 (14.9)	766 (14.3)	162 (5.8)	749 (7.8)	0	0	0	0
Dominican Republic	0	0	49 (1.4)	279 (1.6)	0	0	0	0	0	0	0	0	0	0

Ecuador	30 (1.6)	258 (2.6)	0	0	0	0	0	0	0	0	0	0	0	0
Guatemala	0	0	9 (0.3)	85 (0.5)	46 (2.0)	571 (4.7)	0	0	0	0	0	0	0	0
Mexico	0	0	373 (10.6)	2620 (15.2)	203 (8.7)	2439 (20.0)	157 (16.4)	1809 (33.9)	0	0	0	0	0	0
Panama	0	0	13 (0.4)	31 (0.2)	0	0	0	0	0	0	0	0	0	0
Venezuela	72 (3.9)	440 (4.4)	103 (2.9)	552 (3.2)	142 (6.1)	561 (4.6)	97 (10.1)	198 (3.7)	99 (3.5)	200 (2.1)	0	0	0	0
**Middle East**	0	0	410 (11.7)	1906 (11.1)	362 (15.5)	2023 (16.6)	205 (21.4)	1161 (21.7)	285 (10.2)	2065 (21.5)	222 (13.5)	1956 (35.7)	510 (25.5)	1876 (29.8)
Gulf countries[c]	0	0	58 (1.7)	268 (1.6)	88 (3.8)	369 (3.0)	0	0	0	0	0	0	0	0
Iran	0	0	0	0	199 (8.5)	808 (6.6)	0	0	0	0	39 (2.4)	399 (7.3)	160 (8.0)	403 (6.4)
Iraq	0	0	0	0	0	0	0	0	0	0	84 (5.1)	167 (3.0)	19 (1.0)	33 (0.5)
Jordan	0	0	0	0	0	0	0	0	17 (0.6)	296 (3.1)	12 (0.7)	286 (5.2)	41 (2.1)	309 (4.9)
Kingdom of Saudi Arabia	0	0	66 (1.9)	353 (2.0)	72 (3.1)	252 (2.1)	76 (7.9)	203 (3.8)	96 (3.4)	199 (2.1)	0	0	96 (4.8)	245 (3.9)
Kuwait	0	0	0	0	0	0	0	0	0	0	25 (1.5)	190 (3.5)	20 (1.0)	199 (3.2)
Lebanon	0	0	286 (8.2)	1285 (7.5)	3 (0.1)	594 (4.9)	34 (3.5)	582 (10.9)	98 (3.5)	1059 (11.0)	1 (0.1)	595 (10.9)	64 (3.2)	231 (3.7)
Pakistan[b]	0	0	0	0	0	0	0	0	NA	NA	NA	NA	58 (2.9)	208 (3.3)
United Arab Emirates	0	0	0	0	0	0	95 (9.9)	376 (7.0)	74 (2.7)	511 (5.3)	61 (3.7)	319 (5.8)	52 (2.6)	248 (3.9)

[a]Not all countries in each region were included in each wave; [b]Pakistan was listed under ‘Asia’ region in Wave 5 and Wave 6, and under ‘Middle East’ region in Wave 7; [c]Could include countries listed separately in other waves.

NA, not applicable; T1D, type 1 diabetes; T2D, type 2 diabetes

ESM Table 2. Diabetes therapy use in patients with type 1 and type 2 diabetes

	Wave 1 2005	Wave 2 2006	Wave 3 2008	Wave 4 2010	Wave 5 2011–12	Wave 6 2013–14	Wave 7 2016–17
**Type 1 diabetes (N=15,079)**	**(n=1845)**	**(n=3507)**	**(n=2337)**	**(n=958)**	**(n=2789)**	**(n=1643)**	**(n=2000)**
**Healthy diet and exercise plan, n (%)**	-	-	-	546 (58.0)	1863 (68.0)	1033 (64.8)	1135 (58.3)
**GLP-1 RA, n (%)**	-	-	-	15 (1.6)	31 (1.1)	7 (0.4)	10 (0.5)
**Amylin agonist, n (%)**	-	-	-	1 (0.1)	3 (0.1)	0	3 (0.2)
**Insulin, n (%)**	1845 (100.0)	3507 (100.0)	2337 (100.0)	958 (100.0)	2789 (100.0)	1643 (100.0)	2000 (100.0)
Basal alone	223 (12.2)	509 (14.6)	382 (16.4)	96 (11.7)	161 (5.8)	45 (2.7)	62 (3.1)
Prandial alone	55 (3.0)	85 (2.4)	59 (2.5)	19 (2.3)	78 (2.8)	41 (2.5)	77 (3.9)
Premix alone	443 (24.2)	800 (22.9)	422 (18.1)	141 (17.2)	509 (18.3)	318 (19.4)	388 (19.4)
Basal + Prandial	989 (53.9)	1814 (52.0)	1347 (57.9)	534 (65.0)	1885 (67.8)	1118 (68.2)	1332 (66.7)
Other	124 (6.8)	282 (8.1)	117 (5.0)	31 (3.8)	149 (5.4)	117 (7.1)	139 (7.0)
	**Wave 1 2005**	**Wave 2 2006**	**Wave 3 2008**	**Wave 4 2010**	**Wave 5 2011–12**	**Wave 6 2013–14**	**Wave 7 2016–17**
**Type 2 diabetes (N=66,088)**	**(n=9918)**	**(n=17,232)**	**(n=12,210)**	**(n=5343)**	**(n=9603)**	**(n=5479)**	**(n=6303)**
**Healthy diet and exercise plan, n (%)**	-	-	-	2566 (48.9)	5600 (59.8)	3052 (58.5)	3326 (54.7)
**GLP-1 RA, n (%)**	-	-	-	88 (1.7)	118 (1.2)	95 (1.7)	157 (2.5)
**Amylin agonist, n (%)**	-	-	-	6 (0.1)	14 (0.1)	7 (0.1)	30 (0.5)
**OGLD, n (%)**	8021 (81.0)	14,623 (84.9)	10,596 (87.1)	4904 (91.8)	8216 (85.6)	4767 (87.0)	5573 (88.4)
**Class of OGLD**							
Metformin alone, n (%)	-	3258 (18.9)	2517 (21.1)	1246 (23.6)	2342 (24.4)	1459 (26.6)	1638 (26.7)

Sulphonylureas alone, n (%)	-	2371 (13.8)	1331 (11.2)	307 (5.8)	714 (7.4)	306 (5.6)	257 (4.2)
Metformin with sulphonylureas, n (%)	-	6478 (37.6)	4726 (39.6)	2195 (41.6)	3749 (39.0)	2199 (40.1)	2281 (37.2)
Other, n (%)	-	2494 (14.5)	1781 (14.9)	1092 (20.7)	1409 (14.7)	802 (14.6)	1223 (20.0)
**Insulin, n (%)**	3232 (32.8)	5131 (29.8)	3839 (31.5)	1697 (31.8)	3521 (36.7)	2079 (37.9)	2596 (41.2)
No insulin treatment	6627 (67.3)	12101 (70.3)	8329 (68.6)	3646 (70.8)	6082 (63.4)	3400 (62.1)	3707 (58.8)
Basal alone	1043 (10.6)	2336 (13.6)	1876 (15.4)	823 (16.0)	1333 (13.9)	793 (13.5)	845 (13.4)
Prandial alone	90 (0.9)	118 (0.7)	81 (0.7)	32 (0.6)	40 (0.4)	16 (0.3)	41 (0.7)
Premix alone	1547 (15.7)	16.72 (9.7)	920 (7.6)	255 (4.9)	1012 (10.5)	624 (11.4)	931 (14.8)
Basal + prandial	404 (4.1)	790 (4.6)	838 (6.9)	367 (7.1)	1046 (10.9)	650 (11.9)	715 (11.3)
Other	140 (1.4)	194 (1.1)	102 (0.8)	29 (0.6)	86 (0.9)	50 (0.9)	62 (1.0)
**Patients treated with OGLD only, n (%)**	6389 (64.4)	11403 (66.2)	7979 (65.3)	3530 (66.1)	5912 (61.6)	3321 (60.6)	3637 (57.7)
**Patients treated with OGLD + insulin, n (%)**	1632 (16.5)	3220 (18.7)	2617 (21.4)	1374 (25.7)	2304 (24.0)	1446 (26.4)	1936 (30.7)
**Patients treated with insulin only, n (%)**	1600 (16.1)	1911 (11.1)	1222 (10.0)	323 (6.0)	1217 (12.7)	633 (11.6)	660 (10.5)

Eligible population. GLP-1 RA, glucagon-like peptide-1 receptor agonist; OGLD, oral glucose- lowering drug.

Percentages were calculated for patients with available data; these varied by each category/wave

ESM Table 3. Renal function of patients with type 1 and type 2 diabetes (overall and divided by therapy type): W7 data only

**Renal function (eGFR mL min^{-1} 1.73 m^{-2}), %**	**Stage 1 eGFR ≥90**	**Stage 2 eGFR 60–89**	**Stage 3 eGFR 30–59**	**Stage 4 eGFR 15–29**	**Stage 5 eGFR <15**
**% of patients at each stage**					
**Type 1 diabetes (n=2000)**	64.6	24.9	6.8	1.1	2.6
**Type 2 diabetes (n=6303)**	36.8	41.7	17.6	1.7	2.3
OGLD only (n=3637)	40.7	41.6	14.4	1.0	2.3
OGLD + insulin (n=1936)	33.2	43.4	20.5	1.1	1.8
Insulin only (n=660)	26.7	36.1	25.9	6.7	4.6

Eligible population. eGFR, estimated glomerular filtration rate; OGLD, oral glucose-lowering drug

Percentages were calculated for patients with available data; these varied by each category/wave

ESM Table 4. Hypertension, dyslipidaemia and associated therapy use in patients with type 1 and type 2 diabetes

	Wave 1 2005	Wave 2 2006	Wave 3 2008	Wave 4 2010	Wave 5 2011–12	Wave 6 2013–14	Wave 7 2016–17
**Type 1 diabetes**	**(n=1845)**	**(n=3507)**	**(n=2337)**	**(n=958)**	**(n=2789)**	**(n=1643)**	**(n=2000)**
**Hypertension diagnosis[a]**	416 (22.7)	762 (21.8)	507 (21.8)	214 (22.4)	590 (21.2)	367 (22.4)	396 (19.9)
**Patients treated for hypertension[b]**	394 (94.9)	731 (95.9)	491 (96.8)	210 (98.1)	571 (97.1)	357 (97.3)	375 (95.2)
**Dyslipidaemia diagnosis[a]**	408 (22.6)	808 (25.0)	572 (26.1)	320 (34.7)	617 (23.0)	379 (24.2)	502 (28.9)
**Patients treated for dyslipidaemia[c]**	260 (63.9)	583 (72.4)	449 (78.8)	261 (81.6)	469 (76.3)	283 (74.9)	367 (77.8)
**CV treatments**							
ACEi[d]	256 (65.0)	442 (61.0)	243 (49.5)	87 (41.6)	342 (60.2)	231 (64.7)	208 (55.9)
ARB[d]	71 (18.0)	201 (27.7)	195 (39.7)	100 (47.8)	180 (31.7)	102 (28.6)	114 (30.6)
CCB[d]	91 (23.1)	186 (25.7)	140 (28.5)	59 (28.2)	120 (21.1)	76 (21.3)	67 (18.0)
Diuretics[d]	121 (30.7)	212 (29.2)	120 (24.4)	53 (25.4)	175 (30.8)	106 (29.7)	84 (22.6)
Statins[e]	202 (77.7)	475 (81.8)	390 (86.9)	219 (83.9)	416 (88.7)	256 (90.5)	333 (90.7)
	**Wave 1 2005**	**Wave 2 2006**	**Wave 3 2008**	**Wave 4 2010**	**Wave 5 2011–12**	**Wave 6 2013–14**	**Wave 7 2016–17**
**Type 2 diabetes**	**(n=9918)**	**(n=17,232)**	**(n=12,210)**	**(n=5343)**	**(n=9603)**	**(n=5479)**	**(n=6303)**
**Hypertension diagnosis[a]**	6029 (60.9)	10,681 (62.5)	7422 (61.0)	3254 (61.1)	6315 (66.0)	3623 (66.4)	4166 (66.2)
**Patients treated for hypertension[b]**	5787 (96.2)	10,344 (96.9)	7206 (97.3)	3195 (98.2)	6193 (98.3)	3555 (98.5)	4074 (98.0)
**Dyslipidaemia diagnosis[a]**	4844 (49.3)	9609 (60.2)	7267 (62.5)	3485 (67.2)	5855 (63.4)	3449 (64.8)	3970 (68.9)

**Patients treated for dyslipidaemia[c]**	3785 (78.4)	7866 (82.0)	6275 (86.7)	3051 (87.7)	5170 (88.7)	3094 (90.0)	3552 (91.1)
**CV therapies**							
ACEi[d]	3222 (55.7)	5100 (49.5)	3171 (44.0)	1228 (38.6)	2939 (47.7)	1678 (47.4)	1668 (41.1)
ARB[d]	1400 (24.2)	3325 (32.3)	2888 (40.1)	1532 (48.1)	2445 (39.7)	1397 (39.5)	1770 (43.6)
CCB[d]	1811 (31.3)	3280 (31.8)	2202 (30.6)	850 (26.7)	1655 (26.8)	1125 (31.8)	1384 (34.1)
Diuretics[d]	1834 (31.7)	3325 (32.3)	2093 (29.0)	986 (31.0)	2327 (37.7)	1393 (39.4)	1567 (38.6)
Statins[e]	2968 (78.6)	6273 (80.1)	5154 (82.1)	2512 (82.3)	4558 (88.2)	2819 (91.1)	3365 (94.7)

[a]Diagnosis recorded as 'Yes/No' by the treating physician; [b]Of the patients diagnosed with hypertension; [c]Of the patients diagnosed with dyslipidaemia; [d]Of the patients treated for hypertension; [e]Of the patients treated for dyslipidaemia.

ACEi, angiotensin-converting enzyme inhibitor; ARB, angiotensin II receptor blocker; CCB, calcium channel blocker; CV, cardiovascular

Percentages were calculated for patients with available data; these varied by each category/wave
